# Supplementary material for: Association of Antioxidant Vitamins A, C, E and Carotenoids with Cognitive Performance over Time: A Cohort Study of Middle-Aged Adults
Source: Nutrients. 2020 Nov 20;12(11):3558. doi: 10.3390/nu12113558 (PMC7699702; doi:10.3390/nu12113558)
Supplement: Supplementary file 1 [file nutrients-12-03558-s001.pdf]

ONLINE SUPPORTING MATERIALS

Method S1: Description of cognitive tests, literacy and the CES-D

Mini-Mental State Examination (MMSE)

The MMSE [1] is a cognitive screener that captures global cognitive functioning by briefly measuring orientation, concentration, immediate and short-term memory, language and constructional praxis. Scores range from 0 to 30. Higher scores suggest better cognitive function.

California Verbal Learning Test (CVLT)

The CVLT [2] is a verbal learning and memory test that includes a 16-item word list. A modified version of the CVLT was used with three, as opposed to five, learning trials. Cued recall was not administered. To capture verbal learning and memory, CVLT outcomes variables were total correct score for List A (learning) and List A long-delay free recall (memory). The learning score ranged from 0 to 48 and the memory score ranged from 0 to 16. Higher scores indicate better verbal learning and memory. A more comprehensive description of CVLT can be found elsewhere [2].

Benton Visual Retention Test (BVRT)

The BVRT [3] is a measure of nonverbal memory and visuo-constructional abilities. Administration A, Form D was used. A modified error scoring system based off the BVRT manual was used to guide two trained examiners in scoring the BVRT. Resolution of discrepancies in scoring were attempted by the two examiners, however, if a consensus could not be achieved, MKT, a research psychologist, provided the score. The outcome variable was total errors, with higher values indicating lower visual memory scores.

Digit Span Forward and Backward (DS-F and DS-B)

The Wechsler Adult Intelligence Scale, Revised[4] Digit Span Forward and Backward primarily capture attention and working memory, a component of executive function. The tests were administered according to the manual's instructions. The outcome variable was the total score, which was the total number of correct answers for each test.

Category Fluency

Category fluency[5,6] is a measure of semantic verbal fluency, where participants are asked to generate as many animals as possible within a 60 second duration. Higher scores indicate better category fluency. The outcome variable was the total number of correctly generated words (i.e., words that were *not* intrusions and perseverations).

Brief Test of Attention (BTA)

For the BTA [7], a test of divided auditory attention, the examiner administered up to 10 trials of letters and numbers (4-18 items) that increased in length with each trial. Only the numbers portion of the test was administered. For each trial, participants were asked to disregard the number of letters read, while tracking how many numbers were recited. They were also told to keep their hands in fists to avoid finger counting. The outcome variable was the total number of correct trials.

Trail Making Tests A and B (TRAILS A and B)

The Trail Making Tests A and B[8] primarily capture attention and executive functioning, respectively. The main executive function subdomain that TRAILS B captures is set-shifting and

cognitive control. Both trials also measure visuo-motor scanning and processing speed. Participants were asked to draw a line between consecutive numbers (TRAILS A) and alternate between numbers and letters (TRAILS B) as quickly as they could. They were informed that they were being timed. The examiner pointed out errors that were then corrected by the participant. Errors were captured via increased time. Scores for TRAILS A and B reflected seconds to completion, where higher scores indicate poorer performance.

Clock Drawing Test – Clock to Command (CDT)

The Clock Drawing Test [9] is a measure of visuo-spatial abilities, that also captures elements of memory and executive function. Participants are instructed to draw a clock, put in all of the numbers, and set the hands to 10 minutes past 11. Performance is based off correct drawings of the clock face (0-2), numbers (0-4) and hands (0-4). Scores ranged from 0 to 10, with higher scores indicating better performance. Participants who did not score a perfect score on the command portion of the test were also asked to copy a clock with the hands set to 10 minutes after 11.

Wide Range Achievement Test – 3rd Edition: Word and Letter Reading Subtest (WRAT)

The WRAT Word and Letter Reading Subtest [10] is a test of reading ability that is often used as a proxy for literacy and quality of education. Participants were instructed to correctly read a list of 50 words that increased in difficulty. If the first five words were not correctly pronounced, letter reading was also administered. Standard instructions were used with the tan form. The outcome variable used was the total number of correctly pronounced words.

Center for Epidemiological Studies Depression Scale (CES-D)

The CES-D [11] is a 20-item measure of depressive symptomatology. Participants are asked to consider the frequency and severity of their symptoms over the last week. Scores ranged from 0 to 60. Scores of $\geq$16 indicated significant depressive symptoms and scores of $\geq$20 indicated a clinically significant amount of depressive symptoms.

Method S2: Mixed-effects regression models

The main multiple mixed-effects regression models can be summarized as follows:

	**Multi-level models** vs. **Composite models**		
**Eq. 1.1-1.4**	$Y_{ij} = \pi_{0i} + \pi_{1i} Time_{ij} + \varepsilon_{ij}$	$\pi_{0i} = \gamma_{00} + \gamma_{0a} X_{aij} + \sum_{k=1}^{l} \gamma_{0k} Z_{ik} + \zeta_{0i}$ $\pi_{1i} = \gamma_{10} + \gamma_{1a} X_{aij} + \sum_{m=1}^{n} \gamma_{1m} Z_{im} + \zeta_{1i}$	$Y_{ij} = \gamma_{00} + \gamma_{0a} X_{aij} + \sum_{k=1}^{l} \gamma_{0k} Z_{ik}$ $+ \gamma_{10} Time_{ij} + \gamma_{1a} X_{aij} Time_{ij}$ $+ \sum_{m=1}^{n} \gamma_{1m} Z_{im} Time_{ij}$ $+ (\zeta_{0i} + \zeta_{1i} Time_{ij} + \varepsilon_{ij})$

Where Y_{ij} is the outcome (Each cognitive test score measured at v_1 and/or v_2) for each individual "i" and visit "j"; π_{0i} is the level-1 intercept for individual i; π_{1i} is the level-1 slope for individual i; γ_{00} is the level-2 intercept of the random intercept π_{0i} ; γ_{10} is the level-2 intercept of the slope π_{1i} ; Z_{ik} is a vector of fixed covariates for each individual *i* that are used to predict level-1 intercepts and slopes and included baseline age (Age_{base}) among other covariates. X_{ija}, represents the main predictor variable [v_1 total/individual

carotenoids and other antioxidants]; ζ_{0i} and ζ_{1i} are level-2 disturbances; ε_{ij} is the within-person level-1 disturbance. Of primary interest are the main effects of each exposure X_a (γ_{0a}) and their interaction with *TIME* (γ_{1a}), as described in a previous methodolgical paper.[12]

Table S1. Associations of individual carotenoids (or total carotenoids) and other antioxidants (vitamins A, E and C) with v1 cognitive test score and annual rate of change in cognitive performance: Summary of findings with P<0.05

Models with total carotenoids	
**Total carotenoids**	
Effect on v1 Cognitive test score:<br>Total carotenoids, γ_{0a}	CVLT-List A: -0.0000488±0.0000228, p=0.032
Effect on Cognitive test score change:<br>Total carotenoids×Time, γ_{1a}	CDT: -2.58e-06±1.08e-06, p=0.017
**Vitamin A**	
Effect on v1 Cognitive test score:<br>Vitamin A, γ_{0a}	None
Effect on Cognitive test score change:<br>Vitamin A×Time, γ_{1a}	None
**Vitamin C**	
Effect on v1 Cognitive test score:<br>Vitamin C, γ_{0a}	MMSE: -0.00144±0.000692, p=0.037
Effect on Cognitive test score change:<br>Vitamin C ×Time, γ_{1a}	None

**Vitamin E**	
Effect on v1 Cognitive test score:<br>Vitamin E, γ_{0a}	CVLT-List A: +0.106±0.052, p=0.044
Effect on Congitive test score change:<br>Vitamin E×Time, γ_{1a}	TRAILS B: -0.466±0.210, p=0.027
Models with individual carotenoids	
**Alpha-carotene**	
Effect on v1 Cognitive test score:<br>Alpha-carotene, γ_{0a}	None
Effect on Cognitive test score change:<br>Alpha-carotene×Time, γ_{1a}	None
**Beta-carotene**	
Effect on v1 Cognitive test score:<br>Beta-carotene, γ_{0a}	None
Effect on Cognitive test score change:<br>Beta-carotene×Time, γ_{1a}	CDT(-): -0.0000129±6.37e-06, p=0.042
**Lutein+zeaxanthin**	
Effect on v1 Cognitive test score:<br>Lutein+zeaxanthin, γ_{0a}	None

Effect on Cognitive test score change: Lutein+zeaxanthin ×Time, γ_{1a}	None
Beta-cryptoxanthin	
Effect on v1 Cognitive test score: Beta-cryptoxanthin, γ_{0a}	None
Effect on Cognitive test score change: Beta-cryptoxanthin×Time, γ_{1a}	None
**Lycopene**	
Effect on v1 Cognitive test score: Lycopene, γ_{0a}	CVLT-List A (-):-0.0000694±0.0000309, p=0.025
Effect on Cognitive test score change: Lycopene×Time, γ_{1a}	None
**Vitamin A**	
Effect on v1 Cognitive test score: Vitamin A, γ_{0a}	None
Effect on Cognitive test score change: Vitamin A×Time, γ_{1a}	None

**Vitamin C**	
Effect on v1 Cognitive test score: Vitamin C, γ_{0a}	DS-B (-):-0.0004182±0.0001928, p=0.030
Effect on Cognitive test score change: Vitamin C ×Time, γ_{1a}	None
**Vitamin E**	
Effect on v1 Cognitive test score: Vitamin E, γ_{0a}	CVLT-List A (+): +0.1086146±0.0523306, p=0.038
Effect on Congitive test score change: Vitamin E×Time, γ_{1a}	TRAILS B (-): -0.470414±0.2103017, p=0.025

Abbreviations: γ=Fixed effect; AF=Animal Fluency; BTA=Brief Test of Attention; BVRT=Benton Visual Retention Test; CDT=Clock Drawing Test; CES-D=Center for Epidemiologic Studies-Depression; CVLT-DFR=California Verbal Learning Test-Delayed Free Recall; CVLT-List A=California Verbal Learning Test-List A; DS-B=Digits Span-Backward; DS-F=Digits Span-Forward; HANDLS = Healthy Aging in Neighborhood of Diversity across the Lifespan; HS = High school; MMSE=Mini-Mental State Examination; N-3=Omega-3; N-6=Omega-6; PIR = Poverty income ratio; PUFA=Polyunsaturated Fatty Acids; SE = standard error; T = tertile; TRAILS A=Trailmaking Test, Part A; TRAILS B=Trailmaking Test, part B; WRAT-3 = Wide Range Achievement Test, 3rd revision.

[a] The sample selected has complete data on each of the cognitive test scores at visits 1 and/or 2, complete data on exposures and covariates. Vitamins A, C and E were estimated as average of 2 24 hr recalls from HANDLS first-visit (v1: 2004-2009). The same approach was applied to total and individual dietary carotenoid intakes, as well as other dietary variables. All cognitive are in the direction of higher score → better performance with the exception of BVRT (# of errors) and TRAILS A and B (# of sec. to complete).

[b] Mixed-effects linear regression models were conducted with each cognitive test score as the outcome and each of dietary individual carotenoids and vitamins A, C and E as exposures entered simultaneously. Random effects were added to the intercept and TIME (See **Method S2** for notations). Exposures and covariates were interacted with TIME. Minimally adjusted models (**Model 1**) are presented and included age at v1 (in years) centered at 48, sex (Male vs. Female), race (African-American vs. White), poverty status (Below vs. Above Poverty), education (<HS, HS, >HS), WRAT-3 total score (centered at 42) and employment status (Unemployed vs. employed, Missing vs. employed), total energy intake (kcal/d, centered at 2006) and the inverse mills ratio. Findings are summarized in terms of significant interactions for a specific cognitive test score, at type I error of 0.05 and the direction of the parameter point estimate: +: positive; -: negative.

All cognitive test scores are in the direction of higher score → better performance with the exception of BVRT (# of errors) and TRAILS A and B (# of sec. to complete). See **Table 1** for tertile distribution of total carotenoids.

REFERENCES

1. Folstein, M.F.; Folstein, S.E.; McHugh, P.R. "Mini-mental state". A practical method for grading the cognitive state of patients for the clinician. *J Psychiatr Res* **1975**, *12*, 189-198.
2. Delis, D.C.; Freeland, J.; Kramer, J.H.; Kaplan, E. Integrating clinical assessment with cognitive neuroscience: construct validation of the California Verbal Learning Test. *J Consult Clin Psychol* **1988**, *56*, 123-130.
3. *Revised visual retention test (fifth edition)*. Benton, A.L., Ed.; The Psychological Corportation: New York, 1974.
4. Wechsler, D. *WAIS-R manual.*; The Psychological Corporation: Cleveland, 1981.
5. Morris, J.C.; Heyman, A.; Mohs, R.C.; Hughes, J.P.; van Belle, G.; Fillenbaum, G.; Mellits, E.D.; Clark, C. The Consortium to Establish a Registry for Alzheimer's Disease (CERAD). Part I. Clinical and neuropsychological assessment of Alzheimer's disease. *Neurology* **1989**, *39*, 1159-1165.
6. Morris, J.C.; Mohs, R.C.; Rogers, H.; Fillenbaum, G.; Heyman, A. Consortium to establish a registry for Alzheimer's disease (CERAD) clinical and neuropsychological assessment of Alzheimer's disease. *Psychopharmacol Bull* **1988**, *24*, 641-652.
7. Schretlen, D.; Bobholz, J.H.; Brandt, J. Development and psychometric properties of the Brief Test of Attention. *Clinical Neuropsychologist* **1996**, *10*, 80-89.
8. Reitan, R. *Trail Making Test: Manual for Administration and Scoring*; Reitan Neuropsychological Laboratory: Tucson, AZ, 1992.
9. Rouleau, I.; Salmon, D.P.; Butters, N.; Kennedy, C.; McGuire, K. QUANTITATIVE AND QUALITATIVE ANALYSES OF CLOCK DRAWINGS IN ALZHEIMERS AND HUNTINGTONS-DISEASE. *Brain and Cognition* **1992**, *18*, 70-87.
10. Wilkinson, G.S. *Wide Range Achievement Test–Revision 3*; Jastak Association: Wilmington, DE, 1993.
11. Nguyen, H.T.; Kitner-Triolo, M.; Evans, M.K.; Zonderman, A.B. Factorial invariance of the CES-D in low socioeconomic status African Americans compared with a nationally representative sample. *Psychiatry research* **2004**, *126*, 177-187, doi:10.1016/j.psychres.2004.02.004.
12. Blackwell, E.; de Leon, C.F.; Miller, G.E. Applying mixed regression models to the analysis of repeated-measures data in psychosomatic medicine. *Psychosom Med* **2006**, *68*, 870-878, doi:01.psy.0000239144.91689.ca [pii]

10.1097/01.psy.0000239144.91689.ca.
